# Editorial: The clinical role of auditory implants

**DOI:** 10.3389/fneur.2022.951692

**Published:** 2022-08-30

**Authors:** Maurizio Barbara, Dan Jiang

**Affiliations:** ^1^Department of Neuroscience, Mental Health and Sensory Organs, ENT Unit, Sapienza University Sant'Andrea University Hospital, Rome, Italy; ^2^Skull Base and Auditory Implants, Guy's and St Thomas NHS Foundation Trust, London, United Kingdom

**Keywords:** hearing loss, auditory rehabilitation, bone conducting device, active middle ear implant, ear surgery

Hearing loss is the most common form of sensory impairment in humans, affecting people of all ages. The most recent Global Burden of Disease Study revealed that deafness is the fourth leading cause of years lived with disability and its prevalence continues to grow. Deafness has serious negative consequences for the individual and society, such as difficulties in communication leading to social isolation and withdrawal. It affects mental health and wellbeing, causing depression and anxiety. It is a barrier to independent living and has been linked to dementia. The primary clinical management intervention for people with hearing loss is hearing aids, but not all people with a measurable form of hearing loss could use a hearing aid. The majority (80%) of adults aged 55–74 years, who would benefit from a hearing aid, do not use them. Furthermore, many people who are issued a hearing aid do not wear it (1). Although there is no evidence that hearing aids improve the listening effort of people with hearing loss, there are other reasons that may also influence their uptaking and compliance. Discomfort and occlusion sensations are two of the main complaints of daily use. Recurrent infection of the external and/or middle ear is a contraindication to the use of conventional hearing aids. People also complain about the lack of clarity of sound delivered through conventional hearing aids.

Over the last three decades, the cochlear implant (CI) has become the most successful sensory prosthesis worldwide for the rehabilitation of severe to profound deafness, as it provides open-set speech understanding in majority of the patients, and some patients with CI can also use the telephone (2). Since the introduction of middle ear implantable hearing (MEI) devices and bone conduction implantable hearing (BCI) devices in the 1990s, they also have become acceptable alternatives to conventional hearing aids in selected patients to address associated problems (3). In contrast to CI, which restores the loss of inner hair cell function by transforming the acoustic signal into electrical stimuli for the activation of auditory nerve fibers, these devices stimulate the cochlea by vibrating the skull bone (BCI) or the ossicles/inner ear fluid (MEI) in those with conductive hearing loss, moderate sensorineural hearing loss, or mixed hearing loss.

Despite the huge clinical success of the CI, clinicians still continue to face the challenge of expanding CI indications to allow more people who cannot receive the benefits of conventional hearing aids to be able to receive CI and of optimizing the outcomes in CI users with inner ear or auditory nerve anomalies. A large body of research demonstrating the efficacy of MEI/BCI devices and their clinical safety and shortfalls has been published to date. Despite their clinical potential, they have not been widely used in clinical practice at present, especially in the case of MEI. Only a few centers in the world routinely consider them when counseling patients who are hearing impaired.

The current Research Topic edition included seven timely articles covering a wide range of scientific topics associated with auditory implantable devices. It offers an in-depth look at some of the most relevant issues related to their clinical use. Two articles discussed the challenges posed by cochlear implantation, and five articles reviewed some of the newest research outcomes on active implantable devices in the middle ear.

In a review article by Jenkins et al., the authors examined the existing evidence on the use of round window stimulation to manage patients with mixed hearing loss, while active vibration of the ossicular chain/or stapes is not an option due to existing ear diseases. They elegantly reviewed the basic and clinical research on the mechanism of direct round window membrane stimulation and the validation of its clinical use. The authors recognized the early pioneering research of Colletti et al. (4) on floating mass transducer round window vibroplasty and its clinical shortfalls due to unstable coupling and the subsequent research by many clinicians to improve the reliability of the intervention. They conclude that, while many points remain controversial regarding the parameters of the surgical techniques and actual changes that can occur in forward vs. retrograde stimulation, clinical outcomes have been excellent overall in a population with very little, or available to improve, inner ear stimulation. They also recognized that the lack of a worldwide reimbursement mechanism will continue to prevent future production and advancements of these devices using such technologies.

Another review article, by Shohet and Bibee, summarized the evidence supporting the notion that a fully implantable active middle ear implant, which can provide full-time hearing amplification to those with moderate to severe sensorineural hearing loss, is a viable option for patients who are unable or unwilling to use conventional hearing aids. The article covers an array of wider issues from device characteristics, candidacy, surgical consideration, and programming. The authors outlined the outcome data from most pre- and post-FDA approval clinical studies on this device, which showed better outcomes on objective measures compared with conventional hearing aids, as determined by speech recognition scores, and recognized variable subjective outcomes among these studies. Also, in this article, the lack of a reimbursement mechanism is seen as a major hurdle to its clinical adoption.

Continuing along the lines of the fully implantable active middle ear implant, Monini et al. reported the outcomes of a long-term (4–12 years) follow-up study in which 43 patients implanted with one fully implantable active middle ear implant from a single center were included in the present study. The study found that, after the initial 4 years of post-implantation, some of the patients showed a significant worsening of bone conduction threshold relative to the baseline threshold in the implanted ear as well as a significant decrease when comparing the implanted ear to the contralateral non-implanted ear. The authors attributed the asymmetrical deterioration of the bone conduction threshold to greater energy delivery to the inner ear over time through direct coupling of the device to the stapes. Despite the deterioration of hearing in the implanted ears, the functional gain observed at the initial activation remained constant at follow-up, suggesting an extension of the efficacy of this device in more severe forms of sensorineural hearing loss.

Two articles looked at two different aspects of the floating mass transducer vibroplasties of the Vibrant Soundbridge (VSB). Frohlich et al. described and analyzed VSB-evoked auditory brainstem response (ABR) wave-V intensity-latency functions to coupling efficiency, response thresholds, and coupling modality [oval window (OW) placement vs. incus placement and round window (RW) placement]. They found no correlation between VSB-evoked ABR wave-V intensity-latency functions and coupling efficiency, and there is a rather large variance of individual wave-V latencies at the threshold level. However, the slopes of the intensity-latency function were observed to converge to a steady-state latency of 7–8 ms at the stimulation levels between 30 dB and more above the ABR threshold. The authors suggest that the saturation in VSB-evoked ABR wave-V latencies most likely occurred due to the limited dynamic range of the audio processor used for signal transmission so that the analysis of VSB-evoked intensity-latency functions can be useful for the objective assessment of a patient's dynamic range with the Soundbridge, with vibroplasty-evoked ABR threshold being an objective indicator of coupling efficacy.

Under the hypothesis that auditory cues may contribute to postural control in addition to visual, proprioceptive, and vestibular information, Seiwerth et al. reported their study on the influence of VSB and Bone bridge implantation on patients' balance function. They found that 50% of patients with an optimally turned VSB or Bonebridge BCI had a subjectively positive effect on postural control, despite an improvement that could only be shown objectively in a walking task in the trunk sway measurement and in individual changes in stability in a force plate measurement.

In their study, Wei et al. examined the challenges posed by CI in patients with cochlear common cavity anomalies. Using the multiplanar volume reconstruction (MPVR) techniques to reconstruct postoperative computed tomography (CT) of the temporal bone, they analyzed the correlation of the distance between each electrode and the cavity wall, programming parameters, and performance outcomes. The authors conclude that the shorter the distance between the individual electrode and the common cavity wall, the lower the maximum comfortable level of stimulation, which in turn appears to promote better speech outcomes despite the shorter distance between the individual electrodes and the cavity wall and increases the incidence of facial nerve stimulation.

Benitez et al. reported the outcomes of CI in postlingually deafened children and adults with single-sided deafness (SSD). The results of the study showed that cochlear implantation in postlingually deafened adults and children with SSD can achieve a speech perception outcome comparable to cochlear implementation in conventional candidates. Improvements in the spatial hearing were also observed in those patients with short-term deafness.

In summary, it is our opinion that this collection of articles will provide readers with a better understanding of some challenging issues associated with auditory implants, promote future research in this field, and provide clinicians with better information while making decisions about offering these devices to patients and/or optimizing these devices to rehabilitate hearing loss.

## Author contributions

All authors listed have made a substantial, direct, and intellectual contribution to the work and approved it for publication.

## Conflict of interest

The authors declare that the research was conducted in the absence of any commercial or financial relationships that could be construed as a potential conflict of interest.

## Publisher's note

All claims expressed in this article are solely those of the authors and do not necessarily represent those of their affiliated organizations, or those of the publisher, the editors and the reviewers. Any product that may be evaluated in this article, or claim that may be made by its manufacturer, is not guaranteed or endorsed by the publisher.
